# METASTATIC ANGIOSARCOMA PRESENTING AS DIFFUSE ALVEOLAR HEMORRHAGE

**DOI:** 10.4103/0970-2113.44131

**Published:** 2008

**Authors:** SP Rai, MS Barthwal, P Bhattacharya, S Bhargava, M Pethe

**Affiliations:** 1 Department of Respiratory Medicine, Military Hospital (Cardio-Thoracic Centre), Pune., India; 2 Department of Pathology, Military Hospital (Cardio-Thoracic Centre), Pune., India; 3 Department of Radiology, Military Hospital (Cardio-Thoracic Centre), Pune., India

**Keywords:** Diffuse alveolar hemorrhage, Angiosarcoma, Metastatic

## Abstract

Angiosarcoma is a rare malignant neoplasm of the vascular or lymphatic endothelium. Diffuse alveolar hemorrhage is a rare presenting manifestation of angiosarcoma. We describe a case of pulmonary metastasis of angiosarcoma who presented with diffuse alveolar hemorrhage as initial manifestation.

## INTRODUCTION

Diffuse alveolar hemorrhage (DAH) is a life-threatening clinical yndrome characterized by widespread intra-alveolar hemorrhage^1^. Classic clinical features include hemoptysis, anemia, and diffuse radiographic infiltrates. It has diverse etiologies both immune and nonimmune. The defining pathological feature of DAH is the presence or absence of pulmonary capillaritis^2^. Diagnosis is usually made on the basis of clinical, laboratory, and radiologic findings. Neoplastic disease is not generally considered in the differential diagnosis of DAH and it is rarely the presenting manifestation of angiosarcoma^3^. Angiosarcoma is a rare malignant neoplasm of the vascular or lymphatic endothelium that accounts for 2% of all soft-tissue sarcomas^4^. Angiosarcoma in the lung is a rare disorder and is usually attributable to metastasis from primary site such as skin, soft-tissue, heart, breast or liver. Primary pulmonary angiosarcoma is extremely rare and the prognosis of affected individuals is dismal, with most patients dying within months of presentation. Patients with metastatic angiosarcoma usually have an established diagnosis of malignancy and present with hemoptysis and multiple nodules on chest radiographs^5^. We describe a case of pulmonary metastatic of angiosarcoma who presented with DAH as initial manifestation.

## CASE REPORT

A 26-year-old male was admitted with history of recurrent hemoptysis and breathlessness for two months. There were no other constitutional symptoms. Three weeks earlier he was admitted in a peripheral hospital with hypotension, diffuse bilateral opacities on chest radiograph and pericardial effusion and was treated as a case of disseminated tuberculosis (Pulmonary and pericardial) with anti tubercular treatment (ATT) and oral steroids. On physical examination he was averagely built and nourished and afebrile. His blood pressure was 120/80 mm Hg, respiration 18 per minute and pulse was 80 per minute. He had grade II clubbing and few scattered crackles over both infrascapular area. Other systems were normal.

**Fig 1:**
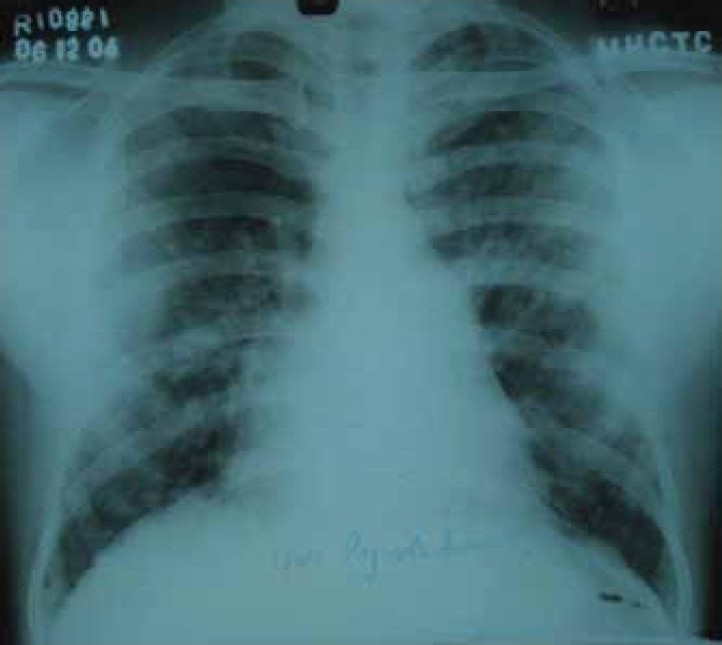
Chest radiograph (PA view) showing diffuse reticulonodular and alveolar opacities of varying sizes from apex to base predominantly in periphery of lung.

**Fig 2:**
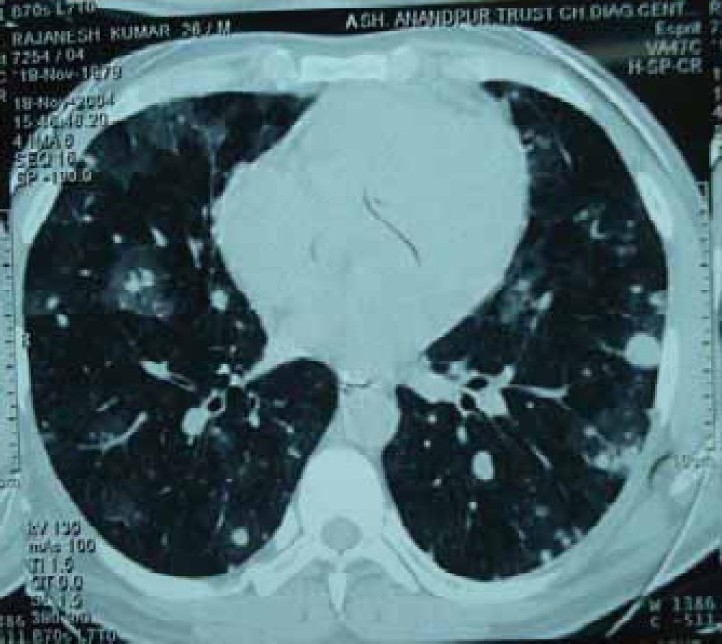
Computed tomographic scan of chest showing multiple patchy ground glass opacities bilaterally with normal intervening lung parenchyma and small nodular opacities scattered in subpleural area

Laboratory investigations revealed hemoglobin: 13 gm/dl, total leukocyte count of 7600/mm3 with polymorphs 64%, lymphocyte 29%, monocyte 2 %, eosinophils 5 % and platelets - 210000 / mm3. His blood sugar, renal functions, liver function tests, electrolytes, urinalysis, bleeding time, clotting time and prothrombin time were normal. Sputum was repeatedly negative for acid fast bacilli. Serology for human immunodeficiency virus (HIV) was non reactor. Peripheral smear showed microcytic hypochromic blood picture. Chest radiograph showed diffuse bilateral alveolar opacities (Figure 1). Contrast enhanced computerized tomographic (CECT) scan of chest showed multiple patchy ground glass opacities bilaterally with normal intervening lung parenchyma and small nodular opacities scattered in subpleural area (Figure 2). Bronchoscopy revealed oozing of blood from all segmental bronchi bilaterally. Bronchoalveolar lavage showed numerous hemosiderin laden macrophages on Perl's stain. Transbronchial lung biopsy showed features of chronic inflammation. Echocardiography showed mild loculated pericardial effusion with no left ventricular dysfunction. The diagnosis of diffuse alveolar hemorrhage was made and he was investigated to rule out any secondary cause. Antinuclear antibodies, antibodies to double stranded DNA, cytoplasmic and peripheral, antineutrophil cytoplasmic antibody (ANCA), rheumatoid factor, immunoglobulin profile, complement level, ultrasound abdomen, 24 hours urinary proteins were normal, ruling out any collagen disorder or renal diseases as a cause of alveolar hemorrhage.

He was managed as a case of idiopathic pulmonary hemosiderosis with prednisilone 60 mg OD for 4 weeks followed by tapering. He showed initially good response with cessation of hemoptysis but subsequently he developed progressive weakness with recurrence of hemoptysis and marked pallor. There was no other obvious source of bleeding from any other site. Repeat chest radiograph showed increase in the diffuse alveolar opacities. His hemoglobin dropped to 3.2 gm/ dl. His repeat coagulation profile and metabolic parameters were normal. He was managed with repeated blood transfusions, broad spectrum antibiotics along with continuation of prednisilone and ATT, but he did not respond. Patient showed progressive deterioration and preterminally (last 4-5 days) developed multiorgan dysfunction in the form of azotemia, deranged liver functions, abnormal coagulation profile and left sided hemiplegia with deterioration of sensorium, suggesting the possible development of septicemia which ultimately led to fatality after 2 months of hospitalization.

**Fig 3:**
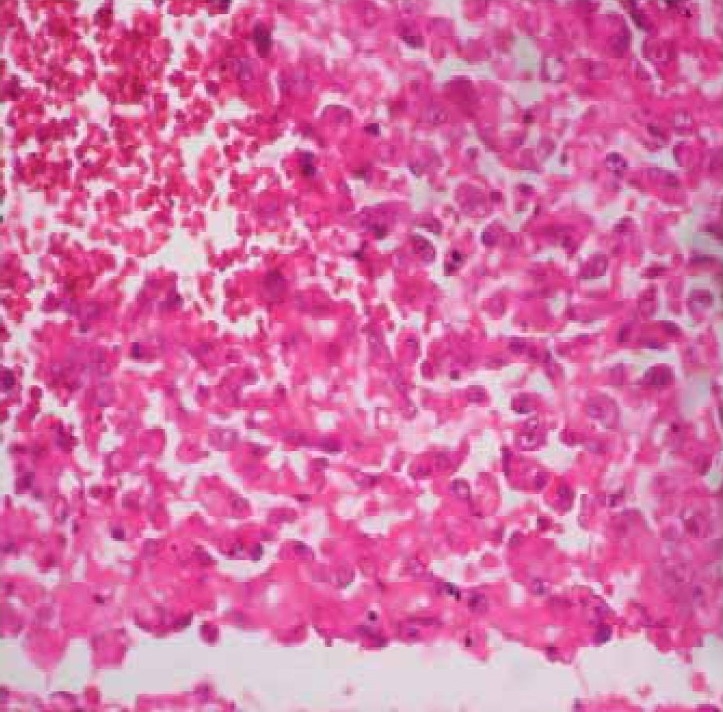
Sections from the lung show large dysplastic spindle cells with hyperchromatic irregular nuclei replacing the lung parenchyma. Adjacent areas of hemorrhage are seen (H&E × 400).

**Fig 4:**
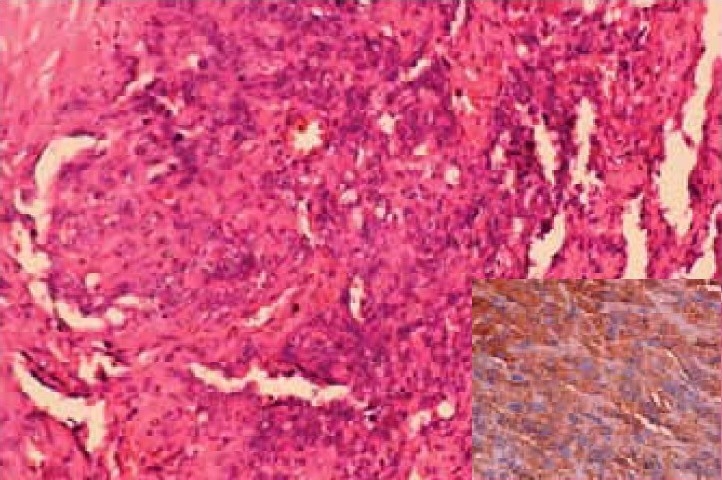
Section from the heart shows large dysplastic spindle cells with hyperchromatic irregular nuclei forming vascular channels and invading into the cardiomyocytes (H&E × 200). Insert: Spindle cells showing CD 31 positivity.

Post mortem showed multiple nodular and hemorrhagic areas in the lungs, liver, stomach, large and small intestines and brain and multiple brownish nodules over pericardial surface. Histopathological examination showed focal hemorrhages in alveolar spaces with many hemosiderin laden macrophages. Dysplastic spindle cells were seen infiltrating into the lung parenchyma (Figure 3). Section from heart showed large dysplastic spindle shaped cells forming vascular channels. The cells showed atypical nuclei with scant cytoplasm. These cells were seen infiltrating into cardiomyocytes (Figure 4). Matastatic lesions of angiosarcoma were also seen in liver, stomach and brain. Immunohistochemistry (CD 31 positivity) confirmed the diagnosis of angiosarcoma (Figure 4 insert).

## DISCUSSION

Angiosarcoma is a rare malignant neoplasm of the vascular or lymphatic endothelium that accounts for 2% of all soft-tissue sarcomas^4^. Angiosarcoma commonly involves skin, soft-tissue, heart, breast or liver. Regardless of their sites of origin, they are particularly likely to metastasize to the lung. Other frequent sites for metastatic spread include the bone, liver, and lymph nodes^5^. Diffuse alveolar hemorrhage is rarely the presenting manifestation of angiosarcoma^3^,^6^ and there are only few case reports. Patients with metastasis of angiosarcoma usually have an established diagnosis of malignancy and present with hemoptysis and multiple nodules on chest radiographs^6^. DAH is a clinical syndrome with diverse etiologies both immune and nonimmune. The defining pathological feature of alveolar hemorrhage (AH) is the presence or absence of pulmonary capillaritis. The ANCA related vasculitis and systemic lupus erythematosus are the commonest causes of immune AH with pulmonary capillaritis, whereas Goodpasture's syndrome and idiopathic pulmonary hemosiderosis are common causes of immune AH without pulmonary capillaritis. The major nonimmune causes of AH are primarily drug induced, or due to hematological malignancy and disorders of coagulation^7^. Clinical features of AH include: dyspnea, fever, hemotypsis, bilateral crackles and pallor. Hypoxemia and bilateral diffuse airspace disease on the chest radiograph with relative sparing of the bases and apices which most often clears within 48 hours after its onset further characterize this syndrome. The diagnosis of AH is confirmed by bronchoalveolar lavage by demonstrating a progressively bloody return on successive aliquots of instilled saline or hemosiderin laden macrophages in the bronchoalveolar lavage fluid^7^. The role of transbronchial lung biopsy is limited in evaluating young adults who present with unexplained DAH^8^. Pulmonary angiosarcoma are usually secondary tumors and only a few primary cases have been reported which has presented with DAH. Transthoracic needle biopsy and transbronchial biopsy have successfully demonstrated pulmonary metastasis in patients with a previously established diagnosis of angiosarcoma, but sampling error can result in misdiagnosis in previously healthy patients like seen in our case. The finding of alveolar hemorrhage without identifiable tumor was considered supportive of a nonneoplastic DAH syndrome but subsequently some of these patients are discovered to have metastatic angiosarcoma^3^,^9^. In a review transbronchial biopsies were nondiagnostic in 5 of 17 patients in which the diagnosis of metastatic angiosarcoma was subsequently made on wedge biopsy or autopsy. The open lung biopsy remains the gold standard for the diagnosis. None of the described patients with metastatic angiosarcoma who presented with DPH were known to have malignancy prior to lung biopsy or autopsy. In 9 out of 17 patients, the primary site was discovered only at autopsy, emphasizing that there is a difficulty in identifying a primary site even after the diagnosis is established^3^. Our patient was diagnosed to have pulmonary metastasis in view of diffuse, bilateral, multifocal lung involvement, without a dominant lesion and heart was the possible primary site.

Diffuse alveolar hemorrhage is an uncommon presenting manifestation of angiosarcoma. Primary or metastatic pulmonary angiosarcoma should be included in the differential diagnosis of DAH especially in young.
